# Implementation of collaborative governance in cross-sector innovation and education networks: evidence from the National Health Service in England

**DOI:** 10.1186/1472-6963-14-S2-P91

**Published:** 2014-07-07

**Authors:** Pavel Ovseiko, Catherine O’Sullivan, Susan Powell, Stephen Davies, Alastair Buchan

**Affiliations:** 1 University of Oxford, Oxford, UK; 2 Thames Valley HIEC, Oxford, UK; 3 Manchester Metropolitan University, Manchester, UK; 4 London School of Hygiene and Tropical Medicine, London, UK; 5 Addenbrooke’s Charitable Trust, Cambridge, UK

## Background

Increasingly, health policy-makers and managers all over the world look for alternative forms of organisation and governance in order to add more value and quality to their health systems. In recent years, the central government in England mandated several cross-sector health initiatives based on collaborative governance arrangements. However, there is little empirical evidence that examines local implementation responses to such centrally-mandated collaborations.

## Materials and methods

Data from the national study of Health Innovation and Education Clusters (HIECs) are used to provide the first comprehensive empirical evidence about the implementation of collaborative governance arrangements in cross-sector health networks in England. The study employed a mixed-methods approach, integrating both quantitative and qualitative data from a national survey of the entire population of HIEC directors (N=17; response rate = 100%), a group discussion with 7 HIEC directors, and 15 in-depth interviews with HIEC directors and chairs.

## Results

The study provides a description and analysis of local implementation responses to the central government mandate to establish HIECs. The latter exemplify cross-sector health networks characterized by a vague mandate with the provision of a small amount of new resources. Our findings indicate that in the case of HIECs such a mandate resulted in the creation of rather fluid and informal partnerships, which over the period of three years made partial-to-full progress on governance activities and, in most cases, did not manage to become self-sustaining without government funding.

## Conclusion

This study has produced valuable insights into the implementation responses in HIECs and possibly other cross-sector collaborations characterised by a vague mandate with the provision of a small amount of new resources. There is little evidence that local dominant coalitions appropriated the central HIEC mandate to their own ends. On the other hand, there is evidence of interpretation and implementation of the central mandate by HIEC leaders to serve their local needs. These findings augur well for Academic Health Science Networks, which pick up the mantle of large-scale, cross-sector collaborations for health and innovation. This study also highlights that a supportive policy environment and sufficient time would be crucial to the successful implementation of new cross-sector health collaborations.

